# Popliteomeniscal fascicles tear with lateral meniscus instability: arthroscopic all-inside technique with two-year follow-up

**DOI:** 10.1186/s13018-025-06452-1

**Published:** 2025-11-19

**Authors:** Giovanni Di Vico, Roberto Simonetta, Alessio D’Addona, Gaetano Correra, Nicola Maffulli, Filippo Migliorini, Donato Rosa

**Affiliations:** 1 Department of Orthopaedics and Trauma Surgery, Clinica San Michele, Maddaloni, Caserta, Italy; 2 Cure Ortopediche Traumatologiche Messina, Messina, Italy; 3 Department of Public Health, Section of Orthopaedics and Trauma Surgery, A.O.U Federico II School of Medicine and Surgery, Via S. Pansini 5, 80131 Naples, Italy; 4 School of Pharmacy and Bioengineering, Keele University Faculty of Medicine, Salerno, United Kingdom of Great Britain and Northern Ireland; 5 Centre for Sports and Exercise Medicine, Barts and The London School of Medicine and Dentistry, Mile End Hospital, 275 Bancroft Road, London, E1 4DG UK; 6 Department of Trauma and Reconstructive Surgery, University Hospital of Halle, Martin-Luther University Halle-Wittenberg, Ernst-Grube-Street 40, 06097 Halle (Saale), Germany; 7 Department of Orthopaedic and Trauma Surgery, Academic Hospital of Bolzano (SABES-ASDAA), 39100 Bolzano, Italy; 8 Department of Life Sciences, Health, and Health Professions, Link Campus University, 00165 Rome, Italy

**Keywords:** Popliteomeniscal, Fascicles, Posterolateral corner, Lateral meniscus, Instability, All-inside, Suture

## Abstract

**Background:**

The popliteomeniscal fascicles (PMFs) connect the lateral meniscus to the popliteal hiatus and provide stability to the lateral meniscus. A high percentage of knees with acute and chronic anterior cruciate ligament (ACL), posterolateral corner injuries and/or hypermobile lateral meniscus (HLM) have concurrent damage to the PMFs. The present study evaluated the outcome of a two-year follow-up of an all-inside arthroscopic procedure to manage tears of the PMFs associated with lateral meniscus instability.

**Methods:**

A total of 11 patients with a tear of the PMFs diagnosed clinically and at MRI underwent an all-inside arthroscopic repair. Patients were enrolled prospectively and evaluated using the Tegner, Lysholm, and IKDC scales for two years.

**Main findings:**

The post-operative MRI revealed that the PMFs had been successfully repaired arthroscopically. According to the subjective IKDC score (*p* < 0.05), the Lysholm score (*p* < 0.001), and the Tegner activity scale (*p* < 0.001), comparing pre-operative and post-operative values, all patients showed significant improvement at the two-year follow-up.

**Principal conclusions:**

Tears of the PMFs predispose the lateral compartment of the knee to chondral lesion, lateral meniscus instability, and progression of osteoarthritis. This study represents the largest series of all-inside repair techniques for PMF disruption. At two-year follow-up, all patients had improved clinically, without any giving way sensation during rotational movements. Most of them had returned to their pre-injury level of activity.

## Introduction

**Fig. 1:**
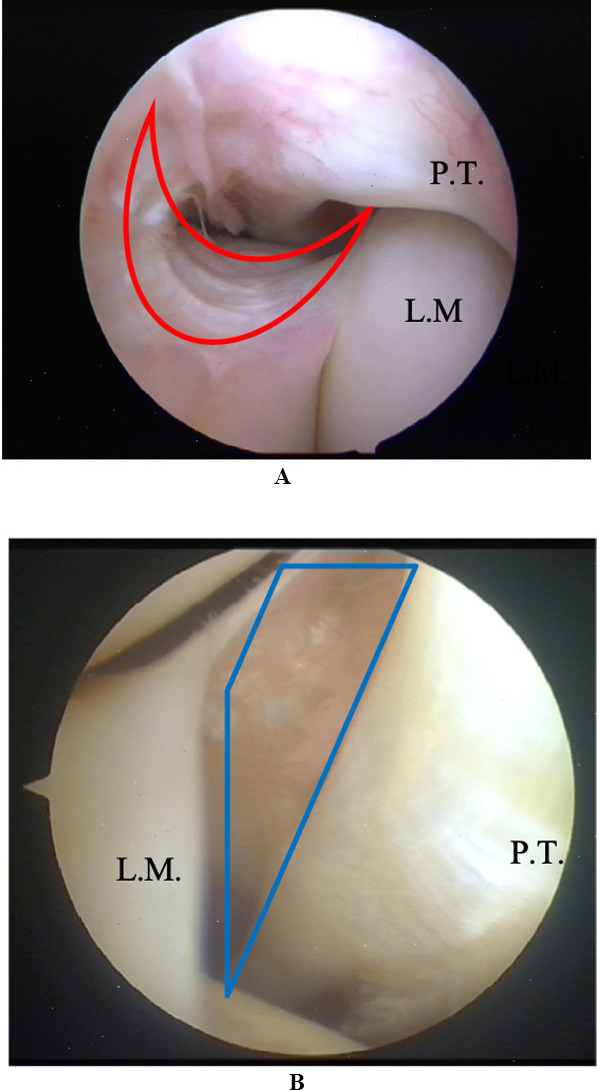
**A**, **B** Popliteomeniscal fascicles. Arthroscopic view with a 70° arthroscope of the PMF bundles, between the lateral meniscus (L.M.) and the popliteal tendon (P.T.): in the upper image, in red the antero-inferior bundle; in the second one, in blue, the postero-superior bundle

The anatomy of the posterolateral corner (PLC) of the knee is complex, and, given the variable injury patterns of this region, controversy and confusion abound [1]. The PLC is composed of several structures, including the lateral meniscal wall, the popliteus muscle and tendon, and the arcuate popliteal ligament. All of them are reinforced by the deep lateral collateral ligament [2]. The popliteomeniscal fascicles (PMFs), one of the several structures of the PLC [1, 3, 4], are composed of a posterosuperior fascicle (sPMF) and an anteroinferior fascicle (iPMF) [5] (Fig. 1A, B). A third inconstant postero-inferior fascicle was also identified in cadaveric specimens [6]. The PMFs connect the lateral meniscus to the popliteal hiatus [7], and provide stability to the lateral meniscus, stabilising the joint during tibial internal rotation and sudden changes of direction [4, 8–10]. The injuries which affect the lateral meniscus lead to an increase in contact pressure and rotational instability, predisposing the joint to osteoarthritis as observed on radiographs [1, 11]. A hypermobile lateral meniscus (HLM) may cause knee pain and a locking sensation during deep knee flexion. One of the most frequent causes of an HLM is thought to be a post-traumatic injury of the PMFs [12–18].

Clinical and imaging diagnosis of lesions in the PMFs is challenging, and MRI using proton density sequences may be useful [8, 19]. Routine knee MRIs on sagittal and coronal planes allow for the detection of such injuries pre-operatively in approximately 60% of cases [8]. The clinical diagnosis is difficult, as most PMFs tears are not isolated injuries [9]. In particular, a high percentage of knees with acute and chronic ACL and/or posterolateral corner injuries have concurrent damage to the PMFs [6, 20]. The risk is that an ACL injury could be incorrectly identified as the sole cause of instability and knee pain [21]. PMFs tears lead to lateral knee pain, painful squatting, and a sensation of locking of the knee [22]. Unfortunately, these clinical signs are not specific to tears of the PMFs. Surgical treatments include open and arthroscopic surgery [19].

We hypothesised that repairing PMFs' tears led to an increase in knee rotational stability, avoiding locking sensation with pain resolution, preventing further lesions (chondral lesion, progression to osteoarthritis of the lateral compartment) and, when performed, protecting the associated surgical procedure (ACL reconstruction). The purpose of the present study is to report the outcome of a two-year follow-up of an all-inside arthroscopic procedure to manage tears of the PMFs associated with lateral meniscus instability.

## Materials and methods

We prospectively enrolled a total of 11 patients from January 2014 to March 2016. All patients were evaluated clinically for PMF tear before the arthroscopic procedure.

**Fig. 2:**
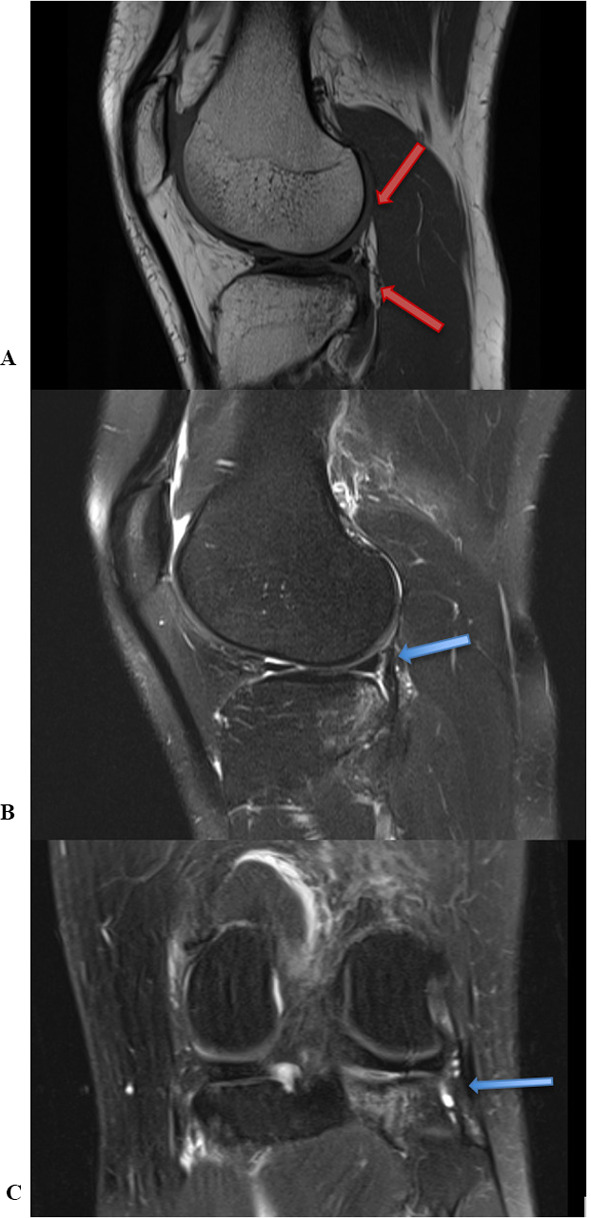
**A**, **B** and **C** Pre-operative MRI. Sagittal (Fig. 2A, B) and coronal (Fig. 2C) views of popliteomeniscal fascicle tears. The red arrows indicate the tear of antero-inferior and postero-superior bundles of PMFs on the sagittal view in T2-weighted sequences. The blue arrow demonstrates the PMF tear in T1 sequences on the sagittal view

Our inclusion criteria were: a tear of the PMF confirmed by clinical examination, with pain at the posterolateral corner (“figure-of-4 test”) [20], sensation of popping, limping, and a feeling of giving way when rotational stresses were imposed with the patient standing. No patient reported actual episodes of giving way. PMF's tears were diagnosed by MRI as the absence of a continuous linear structure and a water-signal interposed between the posterior horn of the lateral meniscus and the joint capsule [5, 23] (Fig. 2A, B and C). Patients with a concomitant ACL tear and/or chondral lesion were not excluded. We excluded patients with concomitant posterior cruciate ligament (PCL) injury, previous meniscal surgery and ligament reconstruction, lateral discoid meniscus, and lateral collateral tear. All patients provided written informed consent, and all procedures were approved by the Local Ethics Committee and performed in accordance with the Declaration of Helsinki. The subjective evaluation scales used were the Tegner Activity Scale, Lysholm Scale, and IKDC. Patients underwent evaluation preoperatively and at 2-year final follow-up.

### Surgical technique

**Fig. 3:**
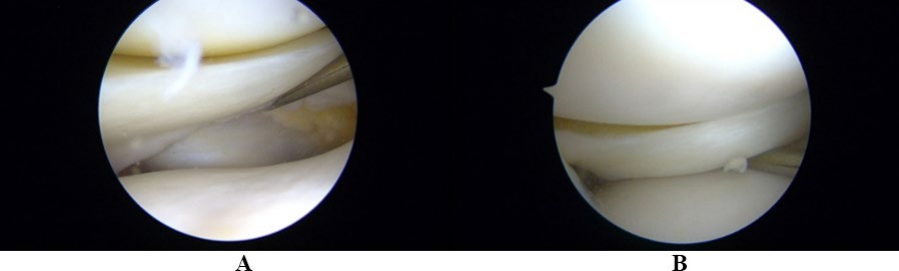
**A**, **B** Intra-operative arthroscopic images of lateral meniscal instability. The instability of the lateral meniscus was demonstrated by probing with an arthroscopic probe

**Fig. 4:**
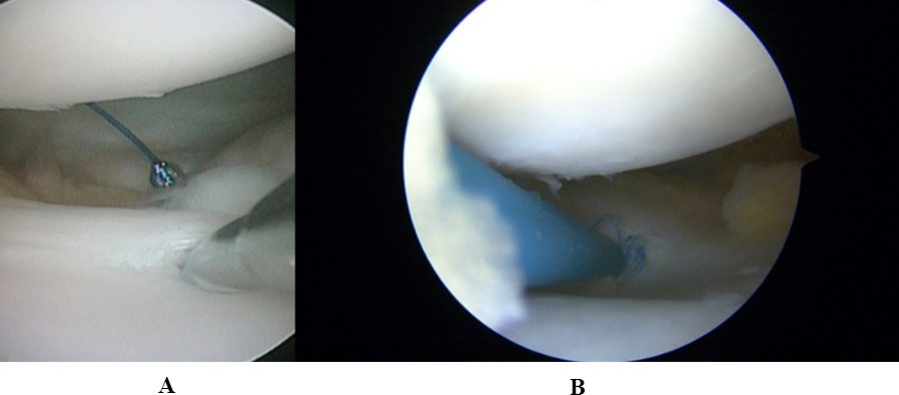
**A**, **B** All inside sutures of PMF tears. After demonstrating the PMF tear and the instability of the lateral meniscus, two or three stitches were placed to stabilise the lateral meniscus, making sure that the knots remained on the capsular side

The patients underwent an arthroscopic repair of the injured PMFs with an all-inside technique. All the procedures were performed by two independent fellowship-trained surgeons (G.D.V. and R.S.). Preoperative antibiotic prophylaxis was administered with 2 g of cefazolin 30 min before performing surgery [24]. With the patient supine with the injured limb in a leg holder, the limb was exsanguinated with an Esmarch band. A thigh tourniquet was inflated to 300 mmHg. A standard diagnostic arthroscopy was performed using standard antero-lateral and antero-medial portals. After having evaluated the intra-articular structures of the knee and excluded other lesions (chondral lesions, foreign bodies, ACL tears, and medial meniscus lesions), the lateral compartment was accurately examined with the knee in the figure-4 position, opening the postero-lateral corner. The stability of the lateral meniscus was checked using an arthroscopic probe. If the meniscus body subluxed towards the medial joint compartment, the meniscus was considered unstable, and the popliteal hiatus was checked for fascicles disruption (Fig. 3A, B).

If a tear of the PMFs was confirmed, a 70° scope was used to visualise the posterior area of the lateral femoral condyle, where it was often possible to identify and address a chondral lesion if present. The suture of the torn PMFs was performed with an all-inside technique using the FasT-Fix device (Smith & Nephew, Andover, MA). Usually, two to three sutures were placed on either side of the popliteal hiatus (Fig. 4A, B), in a vertical fashion between the capsule and the meniscus, making sure that the knots remained on the capsular side.

The surgeon checked that the meniscus was stable, the skin wounds were sutured in a standard fashion, and the knee was placed in a hinged brace locked in full extension for two weeks.

### Postoperative rehabilitation

Patients were positioned in a brace locked in extension for two weeks and allowed weight-bearing as tolerated using crutches. Passive 0–90° range of motion was allowed under physiotherapy supervision and guidance during the first two postoperative weeks, together with single-leg raise. Patients then removed the knee brace, and progressive weight-bearing and active range of motion from 0 to 90° were allowed for the following two weeks. After the first postoperative month, patients were allowed full weight-bearing and full passive range of motion, and they started closed kinetic chain exercises with knee flexion limited to 90°. Running was allowed after the second postoperative month, and return to sports occurred after the fourth postoperative month. Patients were clinically followed at three-month intervals after the first two months for one year, and yearly thereafter.

### Statistics

The *t* test for unpaired data (Graphpad Software, LaJolla, CA, USA) was used to compare the preoperative and postoperative IKDC, Lysholm and Tegner scores. Significance was set at *P* < 0.05.

## Results

Between January 2014 and March 2016, 11 patients met our inclusion criteria and underwent a surgical procedure for popliteomeniscal fascicle repair. Patients’ characteristics are reported in Table 1. Eight patients were male, and three were female, with an average age of 22 years (range 14–35). Six patients were semi-professional athletes (basketball, dancing, and soccer), while the other five practised sports at a recreational level (soccer, volleyball, and basketball). Ten patients were excluded because they reported associated lesions (PCL injury, previous meniscal surgery and ligament reconstruction, lateral discoid meniscus, and lateral collateral tear).

**Table 1:** Patient characteristics

Patient #	Age	Sex	Time from symptoms to surgery	Level of activity	Associated lesions	“Figure-4” test	MRI	Follow-up (months)	Post-op MRI	Return to sport level
1	16	F	24	Semi-pro dancer	LFC lesion	+	+	28	–	Pre-injury level
2	17	M	12	Semi-pro soccer player	LFC lesion	+	+	25	–	Pre-injury level
3	15	M	6	Semi-pro soccer player	None	+	+	26	–	Pre-injury level
4	16	M	10	Semi-pro basketball player	None	+	+	26	–	Pre-injury level
5	17	F	28	Semi-pro dancer	LFC lesion	+	+	24	–	Recreational activity
6	19	M	12	Recreational soccer player	LFC lesion	+	±	24	–	Pre-injury level
7	35	M	6	Recreational basketball player	ACL tear	+	±	26	–	Pre-injury level
8	14	M	2	Semi-pro basketball player	ACL tear	+	+	28	–	Pre-injury level
9	32	M	10	Recreational soccer player	Medial meniscus tear	+	+	25	–	Switch to swimming
10	28	F	7	Recreational volleyball player	ACL tear	+	±	27	–	Pre-injury level
11	34	M	8	Recreational soccer player	ACL tear	+	+	26	–	Pre-injury level

This table reports population data (age, gender, pre-operative level of activity), the duration of symptoms before surgery, pre-surgical assessment (clinical examination using the figure-4 test and MRI scan), follow-up duration in months, assessment at post-operative MRI, and their return to pre-injury level.* LFC* Lateral femoral condyle;* ACL* Anterior cruciate ligament

At presentation, all patients reported a locking sensation and popping, four limped, and all reported tenderness to palpation of the lateral compartment with rotational instability in cutting manoeuvres. A positive “figure-4 test” was reported in all patients. Furthermore, preoperative MRI showed a clear disruption of the PMFs fascicles in the sagittal plane on T2 sequences in 8 patients. In three patients, PMFs tear at MRI was in doubt but with a positive clinical examination. For all patients, the arthroscopic probing of the lateral meniscus confirmed the diagnosis.

In four patients, an associated ACL tear was identified, one patient had a medial meniscus tear, and four of them had sustained a chondral lesion of the lateral femoral condyle not previously reported on pre-operative MRI. All the associated injuries were treated during the same surgery. Most patients had experienced symptoms for more than one year, and the mean time from injury to surgery was 11 months (range, 2–28 months). The mean follow-up duration was 26 months (range, 24 to 28 months). No major complications were recorded at the final clinical examination. Nine patients returned to their pre-injury level of activity, one patient went from semi-professional to recreational activity, and one patient changed his sport from soccer to swimming. Three kinesiophobic patients necessitated prolonged rehabilitation (14 weeks) to achieve knee flexion to 110 degrees. No patients developed a superficial or deep infection.

**Fig. 5:**
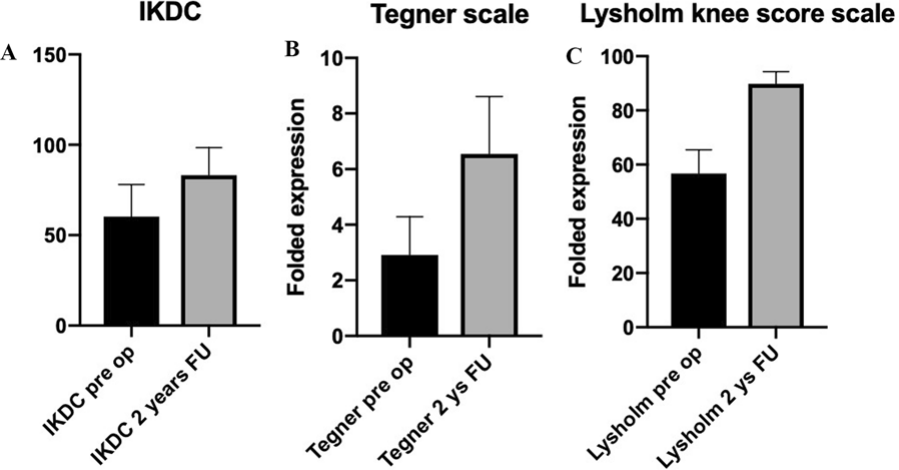
**A**, **B** and **C** Subjective evaluation scales*.* All the scales used showed a significant improvement between pre- and post-operative values at 2 years follow-up. The IKDC scale PMFs and Tegner and Lysholm scores (*P* < 0.001) showed a statistically significant improvement (*P* < 0.05) between preoperative values and 2 years following the index procedure

The average subjective IKDC rose from 60.2 ± 13.5 (range, 27 to 92) preoperatively to 83.1 ± 12 (range, 43 to 100) at last follow-up (*P* < 0.05) (Fig. 5A).

The mean Tegner Activity scale pre-operative score was 2.9 (range 0–5; SD, 1.32), and improved at the final evaluation to 6.5 (range 2–9; SD 2) (*P* < 0.001) (Fig. 5B).

The mean Lysholm knee scoring scale improved post-operatively to 89.8 (range 80 to 96; SD 3.2) from a pre-operative value of 56.72 (range 40–68; SD 8.2) (*P* < 0.001) (Fig. 5C). Three (18%) of the 11 patients reported a good to excellent outcome, and 8 (72%) patients reported a good to fair outcome.

**Fig. 6:**
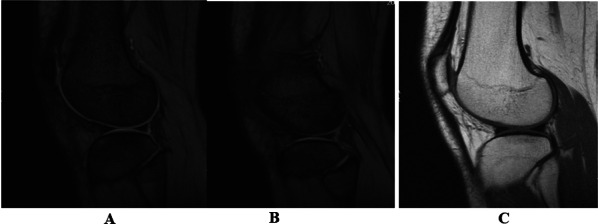
**A**,** B** and** C** MRI post-operative. Successful repair of PMFs both in T1 and T2 in sagittal view

Post-operative MRI showed the successful repair of popliteomeniscal fascicles after their arthroscopic suture (Fig. 6A, B and C).

## Discussion

Posterolateral rotatory instability (PLRI) of the knee is complex and often unrecognised [20]. Providing both a static and dynamic connection between the lateral meniscus and the popliteus tendon, the popliteomeniscal fascicles contribute to controlling the motion of the lateral meniscus during flexion and extension of the knee [1, 9, 21]. Arthroscopic confirmation by probing the lateral meniscus remains the gold standard to confirm the diagnosis of PMF disruption [25].

This is the second-largest case series concerning PMF tear repair, and the largest one regarding the all-inside technique. The major findings of our study are that arthroscopic all-inside repair is a safe and effective treatment for lateral meniscus instability secondary to traumatic disruption of the popliteomeniscal fascicles. Knee function was restored in all patients, allowing most of them to return to sport at their pre-injury level. Two years following the index procedure, the IKDC, Tegner and Lyhsolm outcome evaluation scales showed a significant improvement with full return to pre-injury level of sports in 8 of our 11 patients (72%). Once diagnosed arthroscopically, tears in the PMFs should be surgically repaired [2, 26, 27]. If left untreated, a pathologically hypermobile lateral meniscus may tear secondarily and may require meniscal procedures, with negative consequences to the lateral compartment [28].

Tears of PMFs are present in 80% of patients with an ACL tear or traumatic injury to the PLC, and are associated with a lateral meniscal tear in about 21% of patients at baseline [5, 20, 23, 29]. To prevent residual rotational instability after ACL reconstruction, repairing popliteomeniscal fascicles when a tear is detected and confirmed arthroscopically may be helpful in preserving the ACL graft from re-rupture, thereby avoiding the locking sensation and pain in the lateral compartment associated with lateral meniscus instability. Moreover, subjects with an ACL tear and an isolated PMFs tear experience accelerated cartilage degeneration of the lateral compartment over 2 years [29]. In our case series, four patients reported chondral lesions of the LFC, and for all of them, the time from symptoms to surgery was longer than 12 months. This may indicate that PMFs are an important knee stabiliser and, as far as the surgical approach is considered, more associated lesions could worsen knee function and exacerbate pain. Therefore, if MRI and accurate clinical examination diagnose a PMF tear, and then confirmed arthroscopically by examining and probing the lateral meniscus, we recommend repairing it using an all-inside technique.

Simonian et al. reported satisfying outcomes on three patients treated using an inside-out technique. Complete healing of the lateral meniscus was shown either by postoperative MRI or second-look arthroscopy [18].

Camarillo and Johnson reported on two patients treated with an inside-out repair. While one patient was able to return to sport, the other underwent revision of the repair, and eventually partial lateral meniscetomy [28].

LaPrade and Konowalchuk described an open technique for repairing the lateral meniscus with lesions of the popliteomeniscal fascicles and the popliteus tendon complex. They reported on 6 patients: all were asymptomatic postoperatively and returned to unrestricted activity at an average 3.8-year follow-up [20].

Shin et al. reported an arthroscopic all-inside technique, using a postero-lateral portal through which an arthroscopic suture hook was inserted, with no outcome data. The authors described the triad characterised by mechanical symptoms, hypermobility probing the meniscus during knee arthroscopy, and osteochondral lesion in the posterior portion of the lateral femoral condyle, which strongly suggested a PMF tear [25].

Suganuma et al. described an open repair with an iliotibial band graft to fix the PMFs to the popliteal tendon, restoring their continuity and the stability of the lateral meniscus [20]. This is, however, a demanding non-anatomical reconstruction, although its results seem encouraging.

In a previous preliminary case series, Simonetta et al. described the clinical outcome of six patients treated with the all-inside arthroscopic technique, reporting an excellent clinical and functional outcome with no relapse of pain and a successful return to the pre-injury level of sports activity [27].

Kamiya et al. treated 20 patients using an inside-out technique and reported a statistically significant improvement in the Tegner scale and Lysholm score at postoperative follow-up. Moreover, they used a virtual 3D MRI to assess lateral meniscus hypermobility at active flexion, both pre- and post-operatively [13].

No technique seems superior to the others, probably a result of the lack of head-to-head comparison between the different methods. The present technique, given our skills and experience, provides sound stability to the lateral meniscus and restores normal knee function with the absence of pain or relapse of symptoms at the latest follow-up. Although our results are encouraging, we acknowledge that a limitation of our study is the small number of patients. Furthermore, we did not perform a second-look arthroscopy to assess the healing status and to check the stability of the lateral meniscus. However, it would be ethically untenable to subject asymptomatic patients to a second-look arthroscopy just for documentation purposes. In the future, a larger number of patients will be required to validate our proposed management regimen.

## Conclusion

The popliteomeniscal fascicles contribute to controlling the motion of the lateral meniscus during flexion and extension of the knee. Popliteomeniscal fascicle tears predispose individuals to chondral lesions of the lateral compartment of the knee, lateral meniscus instability, and an early onset of osteoarthritis in the compartment. Our diagnostic algorithm involves a clinical examination, MRI, and arthroscopy to confirm the diagnosis and treat the tear using all-inside suturing. Arthroscopic repair of PMF tears is a safe and effective procedure that provides stability to the lateral meniscus, thereby avoiding subluxation and rotational instability. At two years, patients reported improved symptoms, and most returned to their pre-injury level of sports.

## Data Availability

Data can be provided under reasonable request to Prof Maffulli (n.maffulli@qmul.ac.uk).
