# Correction: Popliteomeniscal fascicles tear with lateral meniscus instability: arthroscopic all-inside technique with two-year follow-up

**DOI:** 10.1186/s13018-025-06603-4

**Published:** 2026-01-14

**Authors:** Giovanni Di Vico, Roberto Simonetta, Alessio D’Addona, Gaetano Correra, Nicola Maffulli, Filippo Migliorini, Donato Rosa

**Affiliations:** 1 Department of Orthopaedics and Trauma Surgery, Clinica San Michele, Maddaloni, Caserta, Italy; 2 Cure Ortopediche Traumatologiche Messina, Messina, Italy; 3 Department of Public Health, Section of Orthopaedics and Trauma Surgery, A.O.U Federico II School of Medicine and Surgery, Via S. Pansini 5, 80131 Naples, Italy; 4 School of Pharmacy and Bioengineering, Keele University Faculty of Medicine, Keele, United Kingdom of Great Britain and Northern Ireland; 5 Centre for Sports and Exercise Medicine, Barts and The London School of Medicine and Dentistry, Mile End Hospital, 275 Bancroft Road, London, E1 4DG UK; 6 Department of Trauma and Reconstructive Surgery, University Hospital of Halle, Martin-Luther University Halle-Wittenberg, Ernst-Grube-Street 40, 06097 Halle (Saale), Germany; 7 Department of Orthopaedic and Trauma Surgery, Academic Hospital of Bolzano (SABES-ASDAA), 39100 Bolzano, Italy; 8 Department of Life Sciences, Health, and Health Professions, Link Campus University, 00165 Rome, Italy; 9 Faculty of Medicine and Psychology, University “La Sapienza” of Rome, Rome, Italy

**Correction to: Journal of Orthopaedic Surgery and Research (2025) 20:1013** 10.1186/s13018-025-06452-1

In this article [1], the given and family names of all the authors were incorrectly structured.

The correct name display should be:

Giovanni Di Vico, Roberto Simonetta, Alessio D’Addona, Gaetano Correra, Nicola Maffulli, Filippo Migliorini & Donato Rosa.

The original article has been corrected.
